# Perspectives in solubility measurement and interpretation

**DOI:** 10.5599/admet.686

**Published:** 2019-04-05

**Authors:** Christel A.S. Bergström, Alex Avdeef

**Affiliations:** 1 Department of Pharmacy, Uppsala University, BMC P.O. Box 580, SE-751 23 Uppsala, Sweden; 2 in-ADME Research, 1732 First Avenue, #102, New York, NY 10128, USA

**Keywords:** Solubility-pH, shake-flask solubility, intrinsic solubility, thermodynamic solubility, Henderson-Hasselbalch equation, supersaturation, pre-nucleation clusters, drug aggregates, drug salts, pharmaceutical cocrystals

## Abstract

Several key topics in solubility measurement and interpretation are briefly summarized and illustrated with case studies drawing on published solubility determinations as a function of pH. Featured are examples of ionizable molecules that exhibit solubility-pH curve distortion from that predicted by the traditionally used Henderson-Hasselbalch equation and possible interpretations for these distortions are provided. The scope is not exhaustive; rather it is focused on detailed descriptions of a few cases. Topics discussed are limitations of kinetic solubility, ‘brick-dust and grease-balls,’ applications of simulated and human intestinal fluids, supersaturation and the relevance of pre-nucleation clusters and sub-micellar aggregates in the formation of solids, drug-buffer/excipient complexation, hydrotropic solubilization, acid-base ‘supersolubilization,’ cocrystal route to supersaturation, as well as data quality assessment and solubility prediction. The goal is to highlight principles of solution equilibria – graphically more than mathematically – that could invite better assay design, to result in improved quality of measurements, and to impart a deeper understanding of the underlying solution chemistry in suspensions of drug solids. The value of solid state characterizations is stressed but not covered explicitly in this mini-review.

## Introduction

For many oral drugs, the rate-limiting step in intestinal absorption is dictated by the kinetics of dissolution of the active pharmaceutical ingredient from the solid form. The dissolution rate is a function of a number of factors, foremost being the aqueous solubility of the drug, which, for ionizable molecules, can be affected by the pH of the medium [1]. Also, depending on the drug that has been introduced, the dissolution may overshoot the saturation concentration and produce a supersaturated solution. The supersaturation is typically achieved when the API is introduced in its amorphous form or in a charged form as a salt. In such a supersaturated solution, drug molecules may self-associate as sub-micellar aggregates/clusters, particularly if they are surface-active or polarizable. For bases introduced as drug salts into an alkaline-pH solution, the charged drug in the supersaturated solution can disproportionate into oil or undergo precipitation into a new amorphous solid, with which charged water-soluble aggregates may co-exist. Given enough time, the oil or amorphous solid (and any other type of aggregates) are expected to undergo phase transformation into a thermodynamically more stable crystalline solid, the steps governed by kinetics of nucleation and crystal growth. For drugs that are practically insoluble in water, dissolution and re-crystallization can be particularly complicated kinetic processes occurring at different interfaces of, *e.g.*, undissolved API or colloidal structures composed of excipients. Aggregation and recrystallization are to date not fully understood and hence may be difficult to predict. About two-thirds of the compounds in the drug discovery pipeline are very poorly soluble in water, and therefore show low and/or erratic intestinal absorption. To increase solubility (and thus absorption), oral drugs are often formulated as salts. However, there are numerous other strategies which have been successfully implemented [2,3].

Several aspects of the above complexity are summarized in this mini-review. It is intended to serve as prologue or accompaniment to an upcoming session on solubility at the IAPC-8 meeting in Split, Croatia, 9-11 September 2019, to be co-chaired by the authors. Since 2009, the International Association of Physical Chemists (IAPC, www.iapchem.org) series of symposia maintained extensive coverage of the topic of solubility measurement, both from solid state and solution perspectives. At the 2015 IAPC-4 meeting, the “*Thermodynamic Solubility Measurement of Practically Insoluble Ionizable Drugs - Case Studies & Suggested Method Improvements*” special session resulted in a ‘white paper’ publication drawing on expert consensus thoughts of researchers from six countries (Hungary, Russia, Serbia, Spain, Sweden, United States) [4]. At the 2017 IAPC-6 meeting, the “*Pharmaceutical Cocrystals -Physicochemical Properties and Formulations*” special session included presentations from internationally-recognized experts in cocrystal solubility-pH measurement. Several publications on the topic emerged out of the meeting. It is anticipated that future sessions will continue to cover solubility methods and strategies, to critically address the different needs in pharmaceutical research, spanning from drug discovery to drug development.

## Brief survey of topics in solubility

Several key topics are briefly summarized below and are illustrated with case studies drawing on solubility determinations as a function of pH. Significant effort was made to find useful examples from the published literature which illustrated principles of solution equilibria. The coverage is not exhaustive, but is considerable in some cases, to address issues of measurement and interpretation of pH dependency in solubility profiles. The value of solid state characterizations is stressed but not covered explicitly in this mini-review.

### Kinetic or thermodynamic?

It’s been more than two decades since Lipinski’s *Rule of 5* was published [5], addressing the problems of poorly soluble compounds. High-throughput microtitre plate methods for determining ‘kinetic’ solubility based on turbidity detection became very popular. Usually, a DMSO solution of the drug is added gradually to an aqueous buffer, until the first signs of solid formation are detected. The resultant precipitate is most often amorphous, up to a hundred times more soluble than that measured by conventional ‘thermodynamic’ (slow-to-medium throughput) methods. Pharmaceutical company databases mostly contain kinetic values. Gradually, it became evident that placing heavy focus on using kinetic data could drive the lead-to-candidate optimization process in the wrong direction: towards making more amorphous-tending compounds, generating problems later on in development [6-8]. Limitations of these early methods can lead to misconceptions and promote the lack of understanding of solubility data [7]. One way to mitigate some of the development-stage risks is to implement ‘miniaturized’ solubility methods (which can produce values closer to those of shake-flask thermodynamic measurements), brought forward *alongside* potency assessments [9]. This is not necessarily easy to do due to the inherently higher costs of measurement, particularly when directed at large compound libraries [10]. Also, such implementation requires some understanding of solubility-pH relationships.

### Brick dust and grease balls

Low-soluble compounds are often colloquially classified as ‘brick dust’ or ‘grease balls’ [11]. The solubility of the former type is mainly limited by a strong crystal lattice, making it difficult for the compound to dissociate from the solid form. Such ‘brick dusts’ are high melting point crystalline solids (T_m_ > 200 °C) in which strong intermolecular bonds within the crystal lattice overcome the competing pull of solvation by water. In contrast, the ‘grease ball’ molecules are limited in their solubility by poor hydration. They typically have high octanol-water partition coefficients (log *P* > 3), and are hydrophobic compounds that form relatively low melting point solids. These compounds are unable to form strong bonds with the water molecules, and hence exhibit high log *P* [12]. Contemporary medicinal chemistry synthesizes compounds as insoluble grease balls rather than brick dust [8,12,13]. Traditional formulation strategies (excipients, solubilizing agents, cyclodextrins, amorphous dispersions, etc.) are likely to improve oral absorption of grease balls, more so than that of brick dust [12]. One may also speculate that many of the ‘brick dust’ molecules never reach the development stage because they are too problematic to work with in assays (*e.g*., pharmacological potency, toxicity and ADME properties). Hence, they are screened out early during the discovery stage.

### Solubility in simulated and human intestinal fluids

A drug’s solubility and dissolution behavior can be investigated in human intestinal fluid (HIF), but such media are difficult to obtain since they require *in vivo* sampling of the fluid. HIF composition is also highly variable [14], both intra- and inter-individually, and it is relatively difficult to work with on the lab bench due to its low buffer capacity. While the variability is important to capture to understand and predict potential population effects, this complexity also makes it more suitable for later stage studies. The development of multiple simulated intestinal fluid (SIF) recipes (FaSSIF, FeSSIF, others) applicable to the early drug development stage has therefore evolved [15]. A recent study by Khadra *et al.* [16] applied a mathematically-rigorous design-of-experiment (DOE) statistical technique to investigate how equilibrium drug solubility can be influenced by seven typical SIF components (sodium taurocholate, lecithin, sodium phosphate, sodium chloride, pH, pancreatin and sodium oleate) within concentration ranges found in HIF. Poorly-soluble acids (naproxen, indomethacin, phenytoin, and piroxicam), bases (aprepitant, carvedilol, zafirlukast, tadalafil), and uncharged drugs (fenofibrate, griseofulvin, felodipine, probucol) were tested. With the exception of pancreatin, all of the factors individually had a significant influence on equilibrium solubility. Differences between acids, bases, and neutral drugs were evident; however, the study also pointed towards (as many other studies also have done) that responses to changes in a solvent, *e.g*., micellar fraction and composition, salt and pH, are often compound specific and there are no generalizing rules or one-size-fits-all solutions.

### Supersaturation, liquid-liquid phase separation, amorphous solid, oil-to-crystal transformations

Buffers can affect the formation of drug aggregates (*i.e.*, molecular clusters). As an example: citrate, succinate, phthalate increase surface activity of phenothiazines; acetate decreases it [17]. Certain surface active bases produce oil when the drug hydrochloride is converted to the free base *in situ* at pH > pH_max_. For example, a 30 μM chlorpromazine hydrochloride solution at pH 9 becomes supersaturated, which then partly separates as oil, which is more soluble than the crystalline free base. In contrast, a 2-5 μM alkaline solution (pH 9-12) prepared from chlorpromazine free base showed no surface activity [17].

**Figure 1.**
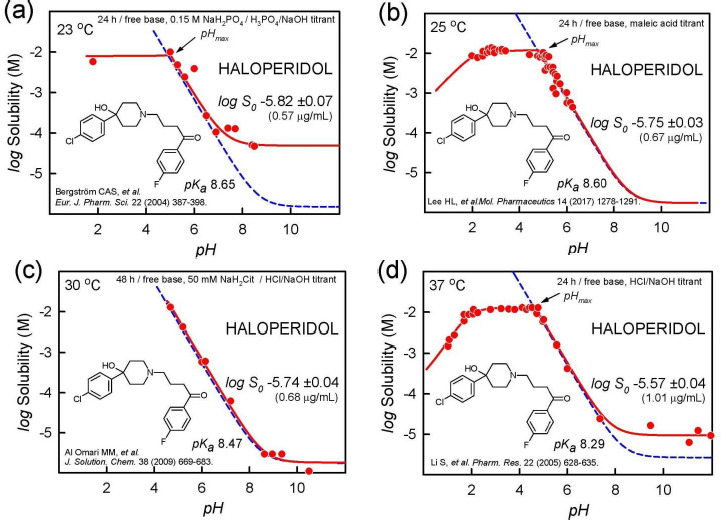
Haloperidol (free base) log S-pH profiles, illustrating alkaline-region distortions from the shape expected from the Henderson-Hasselbalch equation (dashed blue line). The solid red lines are the best fit to the measured data, using the regression analysis program pDISOL-X [22]. See text for further elaboration.

Figure 1 shows log solubility-pH profiles of haloperidol free base at different temperatures and in different buffer media reported by several groups [18-21]. The *dashed* curves refer to the profiles calculated from the simple Henderson-Hasselbalch (HH) equation (not corrected for ionic strength or dilution effects), using the independently-determined p*K*_a_ values appropriate for the temperature. The *solid* curves are calculated using the refined constants from fitting the measured log *S*-pH data (filled circles) to the proposed equilibrium model, using the *p*DISOL-X program (*in-ADME* Research; www.in-adme.com/pdisol_x.html). *S*_0_ refers to the *thermodynamic* intrinsic solubility in pure water of the uncharged form of the dissolved substance in equilibrium with the crystalline solid. Its value is indicated by the lowest value in the *dashed* curve, in the highly alkaline region. The supersaturation in the pH_max_ region noted in the case of chlorpromazine hydrochloride also occurs for the haloperidol mesylate salt (as illustrated in Figs. 3c,d in Ref. [4]). However, it does not occur for the haloperidol free base. Figures 1a,d do show deviations from the theoretical dashed curve (HH) in the alkaline region (pH > 7), where the uncharged species appears to possess elevated solubility.

Several phenomena can account for such a behavior: (i) the neutral (but not the charged) species forms a supersaturated solution; (ii) stable water-soluble uncharged aggregates of haloperidol form; (iii) the added crystalline free base, soon after dissolution, re-precipitate into an amorphous solid phase; (iv) liquid-liquid phase separation (LLPS) takes place, where oil forms as the second liquid phase, in addition to the aqueous phase; and (v) the free-base (but not the charged-form) drug forms a complex with a component of the buffer, thus elevating solubility. Overlapping contributions from the above effects may also play a role.

Without a specific molecular model for the phenomenon of ‘supersaturation’ (i), it is not clear why the extent of distortions is different in each of the frames of Figure 1 (and also in Figs. 3b,c,e in Ref. [4]). Case (ii) – thermodynamically-stable aggregation reaction – is not supported by the data, since the distortion is different in each of the frames. Cases (iii) and (iv) – amorphous solid and oil, resp. – are possible explanations for the distortion. Figure 1c, based on a 48-h equilibration time, shows no distortion in the alkaline region. It could be that the amorphous solid or oil have essentially converted into the thermodynamically most stable crystalline form by then, but not entirely during the 24-h equilibration in Figures 1a,d. The extent of distortion in Figure 1a, compared to Figure 1d, could be indicative of a possible role for drug-phosphate complex formation (v), since 0.15 M phosphate constituted the medium in Figure 1a, but no buffers were in added in Figure 1d.

It is evident from the above examples that solid state characterization would be needed to help decide on the most likely mechanism behind the observed distortions in Figure 1. Changing buffer types in the medium and selecting different equilibration-time approaches [4] would add further insight into the mechanistic basis for the shape distortions in Figure 1.

It is worth noting that a comprehensive analysis of each of the log *S*-pH sets in Figure 1 (e.g., mass action model using all relevant chemical equilibria [22]) yields very similar log *S*_0_ values, showing systematic temperature dependence. Based on van’t Hoff analysis (*e.g.*, Eq. (3) in Ref. [23]), the intercept value of log *S*_0_^25^ at 25 °C is -5.78 ±0.02 and the slope value (enthalpy of solution), Δ*H*_sol_^o^ = 29 ± 6 kJ/mol. Based on the analysis of ten different published studies, the interlaboratory mean value for haloperidol is log *S*_0_^25^= -5.71 ± 0.17 (0.73 μg/mL) for haloperidol [24]. The above 0.17 log unit standard deviation is precisely the value obtained in the averaging of 870 molecules whose solubility was reported in two or more studies, as described below.

A common practice in the literature is to report the measured solubility near pH = p*K*_a_ +/- 2 (for bases/acids, resp.) as the intrinsic value. The above examples suggests that caution is warranted for such a procedure: from Figures 1a,c,d, the mean value would be -5.07 ±0.82 (3.2 ±16 μg/mL), which would not represent the realistic accuracy of the underlying data. A common perception is that reproducibility in solubility measurements is typically not better than 0.7 log unit [25]. The haloperidol example suggests that better reproducibility can be achieved if published data are critically analyzed. The diclofenac example below will further elaborate this.

**Figure 2.**
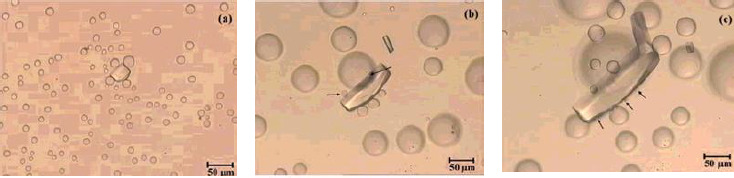
Time sequence of liquid-liquid phase boundary transformation to crystalline phase at 0, 12, and 22 h [26]. Reprinted with permission from S. Veesler, L. Lafferrère, E. Garcia, C. Hoff, Phase transitions in supersaturated drug solution. Org. Proc. Res. Dev. **7** (2003) 983-989. Copyright 2003 American Chemical Society.

The haloperidol discussion suggests that oil or amorphous solid forms in alkaline solutions. Generally, a supersaturated solution forms on initial dissolution. Such a solution can then spontaneously form two liquid phases (aqueous and oil), followed soon after by the appearance of crystals, as shown in the time sequence in Figure 2. Solute is transported from oil drops to crystal via the continuous phase in a solvent-mediated process in which the drops disappear as the crystal grows. After heating and cooling the crystals, LLPS is not regenerated, which further confirms that the LLPS is not a thermodynamically stable phase [26,27].

### Amorphous and crystalline solids

**Figure 3.**
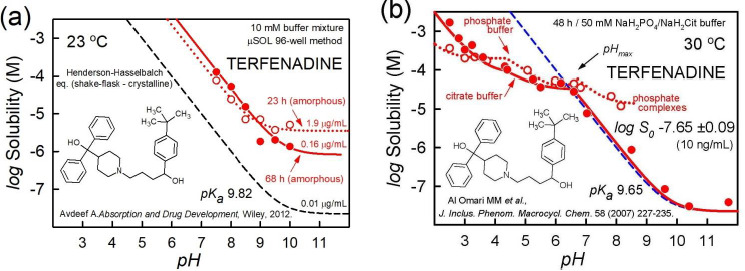
Terfenadine examples of oil/amorphous vs. crystalline phases solubility-pH profiles. **(a)** High-throughput measurement [29]. Dotted curve is best-fit of 23-h equilibration data; solid curve is best-fit of 68-h data. As a visual guide, the dashed curve is the HH curve calculated for crystalline terfenadine, based on the analysis of the data from Al Omari *et al*. (2007) [29], shown in the frame on the right. **(b)** Shake-flask thermodynamic measurements [29]. See text.

High-throughput solubility (HTS) methods, where DMSO solutions of the sample are added to buffer media, generally indicate a measure of the solubility of amorphous solids, with values greater than that indicated by crystalline solids. Amorphous phases are not thermodynamically stable and will undergo crystallization over time. Two amorphous phases may co-exist, or a transient oil phase may appear. An example of this is shown in Figure 3a for the case of terfenadine measurement using the μSOL method (Pion Inc.), with 1 %v/v DMSO in each of the 96-well buffer solutions [28]. The unfilled circles represent solubility values collected at the end of 23-h equilibration, and may suggest the presence of oil *plus* amorphous solid. (Alternatively, the ‘oil’ can be a more energetic amorphous solid co-precipitated with a more stable amorphous form.) The filled circles represent data collected at 68-h equilibration time, and may suggest the sole presence of the more stable amorphous solid. The *apparent* solubility at pH 11 is 1.9 μg/mL for the shorter time. This value appears to be an order of magnitude greater than the analyzed value of 0.16 μg/mL, corresponding to the *apparent* intrinsic solubility of the more stable amorphous solid. Solid state characterizations were not reported for the above HTS measurements.

The dashed curve in Figure 3a represents the thermodynamic solubility of the crystalline solid, 0.01 μg/mL, based on the reported citrate-buffer data of Al Omari *et al*. [29]. The detailed analysis of the latter is shown in Figure 3b. The authors studied the solubility characteristics in 50 mM phosphate (unfilled circles) as well as citrate (filled circles) buffer. The 30 °C shake-flask data at 48-h equilibration appears to be of good quality. The phosphate-containing medium seems to indicate drug-buffer complexation for pH > pH_max_ (not seen in the citrate medium), as evidenced by the elevation in solubility above the dashed HH curve (pH 7-8). The analysis suggests the equilibrium constant *K* = [Tf.TfH^+^.HPO_4_^2-^]/[Tf][TfH^+^][HPO_4_^2-^] = 10^+8.96^ M^-2^ for the monoanionic complex [24]. Between pH_max_ 6.7 and pH 8.3, the complex appears to shed one proton with increasing pH. In both the citrate and phosphate media, at pH below pH_max_, two salts form between the drug and each buffer, consistent with the solid and dotted curves being below the HH dashed curve. The salt solubilities are quite similar for the citrate and phosphate systems.

**Figure 4.**
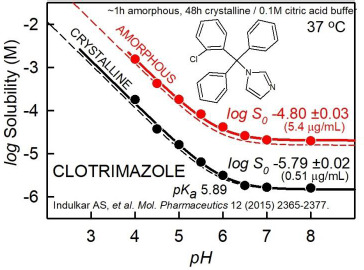
Clotrimazole example of amorphous vs. crystalline phase solubility-pH profiles [30]. See text for details.

Figure 4 shows a simpler example of a single amorphous-phase log *S*-pH profile of clotrimazole, measured at about 1-h equilibration time, compared to the corresponding crystalline phase profile, measured at 48-h equilibration [30]. In both cases, the curves nearly follow the pH dependence calculated by the Henderson-Hasselbalch equation (with a constant vertical displacement between the two), although the amorphous curve shows a slight amount liquid-liquid separation above pH 7, as indicated by a small difference between the solid and dashed upper curves in neutral solution (which is absent in the lower crystalline curves).

### Pre-nucleation clusters (i.e. self-aggregates, sub-micelles, drug-buffer complexes)

Although classical nucleation theory describes drug crystallization from a supersaturated aqueous solution, the understanding of the molecular basis underlying the nucleation steps is still incomplete [31]. The role of pre-nucleation clusters as solute precursors to nucleation and the link between the chemical speciation of homogeneous solutions and the process of phase separation of such clusters was reviewed by Gebauer *et al.* [31]. Sub-micellar pre-nucleation clusters form in many supersaturated solutions: calcium phosphate, amino acids – particularly glycine (see below), and many surface-active drug molecules, such as in phosphate-buffered solutions of chlorpromazine, acetylpromazine, verapamil, orphenadrine, hydralazine, pramoxine, and amiodarone, at pH slightly above that of pH_max_ [21,32]. The buffers in solution critically influence the stability of such clusters. The link between the clusters and the kinetics of crystallization is still work in progress, and could turn out to be a vibrant area for future investigations. It should also be noted that the loss of supersaturation may potentially start at the surface of the dissolving material that is provided in excess and not always from the supersaturated solution [33].

**Figure 5.**
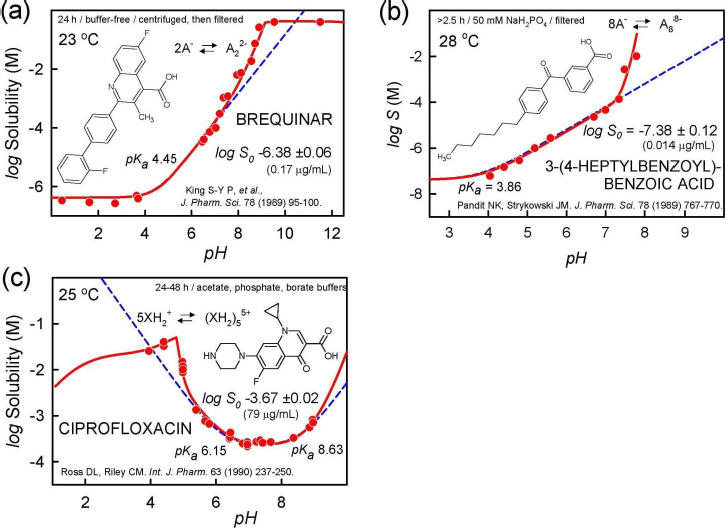
Examples of charged-species aggregation. **(a)** Brequinar appears to form a negatively-charged dimer above pH 7, as indicated by a slope of +2 in the log S-pH plot. Sodium-drug salt precipitates above pH 9 [34]. **(b)** 3-(4-Heptyl-benzoyl)-benzoic acid appears to form an 8-monomer aggregate above pH 7, as indicated by the slope of +8 in the plot [35]. **(c)** Ciprofloxacin cation appears to form a pentameric aggregate below pH 6, as well as a phosphate salt for pH < pH_max_ [36]. The possibility of drug-phosphate complexation has not been ruled out.

Solubility-pH profiles can indicate self-aggregation/cluster formations. In the pH region where the drug is charged and self-aggregation takes place, the slope in the log *S*-pH plot can reveal the order of aggregation [28]. Figure 5 shows three examples indicating formation of charged aggregates. In Figure 5a, brequinar appears to form a negatively-charged dimer at *pH* above 7, as indicated by a slope of +2 in the log *S*-pH plot; the corresponding sodium-drug salt precipitates above pH 9 [34]. Figure 5b shows the surface active molecule, 3-(4-heptylbenzoyl)-benzoic acid, apparently forming a negatively-charged octamer above pH 7, as indicated by the slope of +8 in the plot [35]. The authors proposed that anionic micelles formed, with a critical micelle concentration of about 0.3 mM. Figure 5c shows ciprofloxacin cation appearing to form a pentameric aggregate below *pH* 5 (slope = -5), overlapping with the formation of a phosphate salt at lower pH [36]. (The possibility of the formation of a soluble drug-phosphate complex near pH 5 has not been explored.) Most often, when such sub-micellar charged aggregates form, the distortion in the log *S-*pH curve is most prominent right below the pH_max_ for acids and right above the pH_max_ for bases, as indicated by the examples in Figure 5.

**Figure 6.**
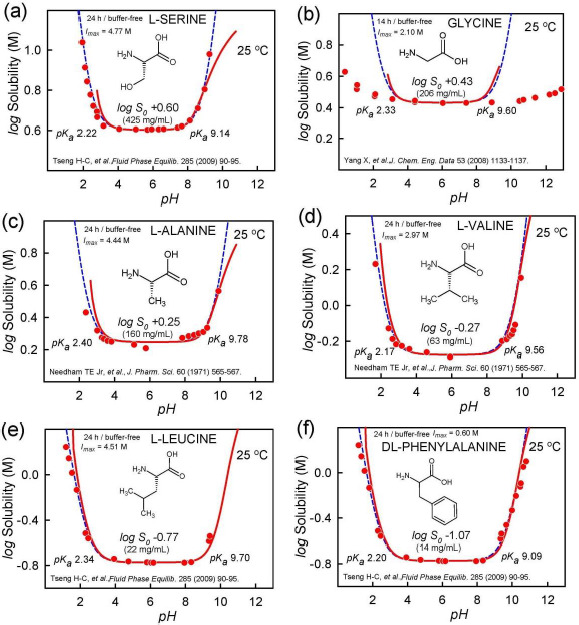
The solubility-pH profiles of amino acids showing departures from the ideal Henderson-Hasselbalch shape. The distortion is particularly extreme in the case of glycine. Since the data for pH < 4 and pH > 8 were not used in the regression analysis, the listed log S_0_ values are ‘apparent.’ The actual values may be lower, especially for glycine.

As discussed by Gebauer *et al.* [31], amino acids, particularly glycine, form sub-micellar pre-nucleation clusters. Figure 6 displays log *S-*pH profiles of six amino acids in buffer-free solutions, where pH was modified with HCl/NaOH [37-39]. Amino acids are highly soluble in water. Consequently, the ionic strength reaches nearly 5 M at the low-pH end of the profile in three of the cases. The pH < 3 and pH > 9 electrode readings may be significantly affected. The symmetrically U-shaped dashed curves in the figures were calculated using the simple HH equation, which does not factor in the effects of ionic strength. The solid curves, which are less symmetrical than the HH curves, are the results of regression analysis where effects of ionic strength were factored in [22]. The *apparent* log *S_0_* values listed in the figures were calculated using only the data in the pH 4-8 interval, with the rest of the points assigned zero weights during the regression analysis. It can be seen that for pH < 3 and pH > 9, the measured points are notably below the *solid* curves. This is extremely so in the case of glycine in Figure 6b. Ordinarily, such distortion would suggest that the zwitterionic form of the molecules self-associate, thus raising the solubility [28]. However, it was not possible to fit the glycine data with an aggregation model that contained a single zwitterionic stoichiometry. At least several overlapping oligomers appear to be necessary to rationalize the nearly flat log *S-*pH profile of polymeric glycine. It may be that glycine forms a high-molecular weight polymer strands in saturated solutions, which persist across a broad swathe of pH.

### Hydrotropic solubilization via cluster formation

**Figure 7.**
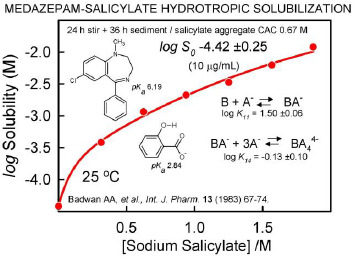
Hydrotropic solubilization. Plot of the total concentration of medazepam in a saturated solution as a function of added sodium salicylate. The solubility data of Badwan *et al*. [42] were fit (using pDISOL-X) to an equilibrium model consisting of the drug forming a complex with four units of salicylate. ‘CAC’ is the abbreviation for critical aggregate concentration.

In 1916, Neuberg [40] coined the term ‘hydrotrope’ for compounds that increase the solubility of sparingly-soluble organic substances, by interacting as ‘co-solubilizers.’ Usually, high concentrations of hydrotropes are necessary to increase solubility of the sparingly-soluble substance. Hydrotropes are thought to form aggregates in solution. Typical examples of hydrotropes include urea, nicotinamide, sodium salicylate, and benzoates with a short alkyl chains, as these molecules can self-associate into small, sub-micellar clusters of 3-4 molecules [41]. A poorly-soluble molecule can be associated with these clusters and become solubilized in the process. Figure 7 shows an example of the hydrotropic solubilization of medazepam by salicylate anions [42]. It would be interesting to explore whether phosphate at high concentrations acts as a hydrotrope.

### Supersolubilization and amorphous dispersions

Parikh and Serajuddin [43] have developed a novel method of preparing solid dispersion by using acid-base interaction in water. Haloperidol was dissolved in *concentrated* aqueous solutions of a weak acid (e.g., malic, tartaric, or citric acid). Each weak acid was selected such that it would not form salts with the drug. The solubility of haloperidol exceeded 300 mg/g aqueous solution of malic acid. This was an extremely high aqueous solubility for haloperidol, considering that its intrinsic aqueous solubility in water as the free base is only 0.7 μg/mL, and the solubility of its salt forms (e.g., HCl, phosphate, maleate) is only about 1-5 mg/mL. Similar solubility enhancements were observed with itraconazole by the investigators. The extraordinary enhancement by acid-base interaction has been called ‘supersolubilization.’ On drying, the drug-containing material is a semisolid and sticky amorphous solid dispersion. Free-flowing powders that can be compressed into tablets are obtained by e.g., adsorbing drug solid dispersions with acids onto porous silica [44].

### Cocrystals enhancement of solubility

Xanthines (e.g., caffeine, theophylline, theobromine) are known to form complexes with many pharmaceutical compounds. In a series of papers (1952-1954) Higuchi, Zuck and Lach [45] studied the xanthine molecular associations formed with simple organic molecules in saturated solutions. Weak caffeine-containing complexes were determined to form, in the decreasing order of stability: p-hydroxybenzoic acid > benzocaine > butylparaben > p-aminobenzoic acid > salicylic acid > benzoic acid > aspirin > o-phthalic acid > benzoate > sulfathiazole > picric acid > sulfadiazine > suberic acid. Such complexes raised the solubility of the xanthine. These classic studies may bear relevance to the selection of ‘coformers’ in cocrystal formation.

A recent and still evolving formulation strategy to increase intestinal absorption is based on using cocrystals [46]. Cocrystals are materials composed of two or more different uncharged molecules (e.g., drug and ‘coformer’) within the same crystal lattice, which are associated by nonionic and noncovalent bonds. The altered solid state properties in cocrystals are generally expected to elevate the drug concentration in a similar way that drug salts do, as the drug is released in a dissolution process. The initially elevated concentration of the drug could lead to increased oral absorption of the drug. The analyses of solubility of salts and cocrystals are similar, since both are multi-component solids. Drug salts are characterized by a solubility product, *K*_sp_^SALT^= [drug][counterion], where the drug is *charged*. Cocrystals are similarly characterized by an equilibrium solubility product, *K*_sp_^CC^ = [drug][coformer], but in contrast to salts, both the drug and the coformer are *uncharged* in the cocrystal. A method to predict *K*_sp_^CC^ and the solubility enhancement of cocrystals, using an approach based on measured drug and coformer intrinsic solubility (*S*_0_^drug^, *S*_0_^cof^), combined with *in silico* H-bond descriptors, has been recently described [47,48]. The latter publications arose out of the IAPC-6 special session on cocrystal solubility.

**Figure 8.**
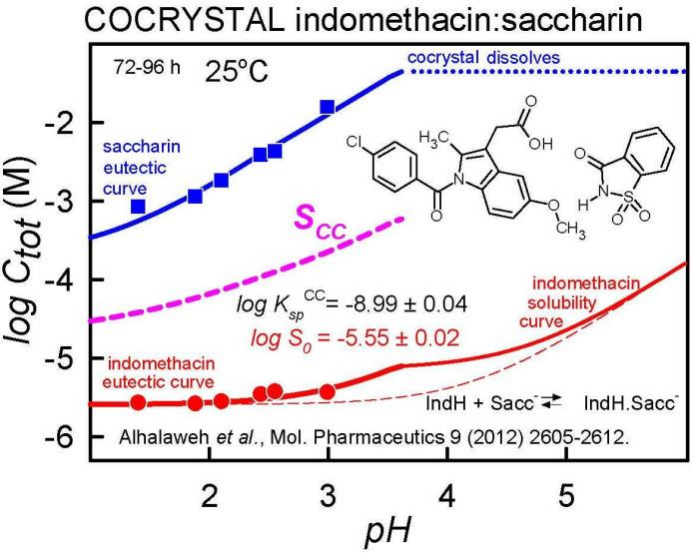
Plot of the total equilibrium concentrations of saccharin (upper solid line, blue), indomethacin (lower solid line, red), and of the maximum possible concentration of the solubilized indomethacin released from the cocrystal (thick dashed curve, S_CC_), as a function of pH. S_CC_ is termed the ‘cocrystal solubility.’ The elevation of the S_CC_ dashed curve above that of the indomethacin equilibrium solid red curve is viewed as the cocrystal ‘solubility advantage.’ The solubility product is no longer satisfied above pH 3.7, so the cocrystal no longer forms above that pH.

Figure 8 shows an example of a solubility profile as a function of pH for the indomethacin:saccharin cocrystal [49]. The points represent the measured total concentrations of the coformer saccharin (blue squares) and the drug indomethacin (red circles) under equilibrium conditions poised at 72-96 h, where both the cocrystal and the free-acid crystalline drug are indefinitely stable co-precipitates below pH 3.7. As the cocrystal partially dissolves below pH 3.7, it releases the solvated free drug at the concentration indicated by the *S*_CC_ thick dashed line. This is the initial supersaturated concentration of the drug. It may not persist long, but while the drug is in the elevated concentration, its intestinal absorption may be significantly enhanced. The Henderson-Hasselbalch curve for indomethacin is indicated by the dashed red curve in the bottom of the drawing. The equilibrium concentration of indomethacin (solid red curve) is above the dashed curve, with the difference peaking at about pH 3.7. This can be explained by the complexation of uncharged drug by the anionic saccharin. The effect diminishes above pH 5, since the concentration of the free drug diminishes due to ionization [47].

### Data quality

A comprehensive study of equilibrium solubility data quality based on the review of over 800 publications was reported in 2015 [50]. Since then, more studies have been examined and a more robust estimate of the true interlaboratory reproducibility can be offered here. Improved estimates [4,50] of interlaboratory reproducibility are better revealed when solubility data are normalized for pH (to produce intrinsic solubility, *S_0_*, derived from water solubility, *S*_w_, or multiple-pH measurements) and temperature (by transforming measurements performed in the range 10-50 °C to the standard value of 25 °C [23]).

**Figure 9.**
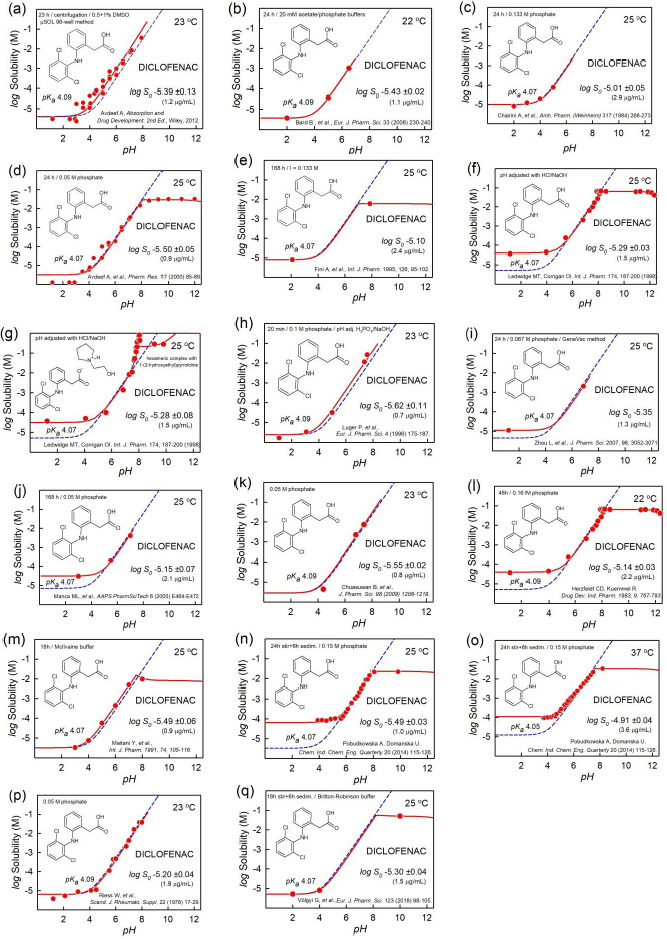
The diclofenac log *S*-pH measurements from 17 publications [28,51-64] reveal a plethora of effects that might lead to poor reproducibility in the determination of the intrinsic solubility. See text for details.

By way of an addendum to the 2015 study, the data-quality study now has 6355 intrinsic solubility entries in the *Wiki-pS_0_* database (*in-ADME* Research), for 3014 different pharmaceutically-relevant molecules (solids at room temperature), drawing on the review of 1325 publications. Of all the entries, 2144 are singletons, generally reported as *S_w_*, but transformed to *S_0_*, and temperature adjusted (if needed) to 25 °C. These single-source results offer no direct indication of interlaboratory reproducibility. That indication comes from the 870 molecules for which solubility was reported from at least two different sources. Some molecules have been studied in surprisingly large number of different laboratories. For example, there were 34 different reports of the solubility of diclofenac found to date. Seventeen of these were measured at several different pH values, and are displayed in Figure 9. The next most-frequently studied molecules appear to be phenytoin, barbital, and ketoprofen with 30, 26, and 24 interlaboratory determinations, respectively. The average interlaboratory reproducibility, based on the curated 870 replicated studies, has been determined to be 0.17 log unit, significantly lower than the interlaboratory reproducibility (~0.7 log unit) suggested in past studies [25]. Many factors can lead to the perception of poor quality of data. Comparing “raw” water solubilities (*S*_w_) determined in unbuffered solutions can be problematic. For example, using inaccurate values of p*K*_a_ to interpret the log *S-*pH data can lead to erroneous intrinsic solubility values. Ambient levels of carbon dioxide can significantly affect measured *S*_w_ values in the cases of sparingly soluble bases. So can unsuspected buffer-drug interactions, aggregate/micelle formations, presence of amorphous solids, supersaturated solutions, differences in the measurement temperature, and other experimental conditions. When an effort is made to critically factor in the possible sources of systematic error, the interlaboratory reproducibility is actually quite good. Still, for some drug molecules, the intrinsic solubility is very uncertain, with standard deviation (SD) values exceeding 0.5 log unit.

In addressing data quality, it needs to be emphasized that standard conditions are essential: *intrinsic* solubility values are to be compared at the designated reference temperature of 25 °C. Mixing different types of solubility and not normalizing for temperature increase the apparent variance between different laboratory results, as noted in the haloperidol example (*cf*., Fig. 1). Although the realistic average interlaboratory reproducibility is relatively good, some low-soluble molecules are still problematic. Table 1 lists the 32 compounds with the poorest reproducibility, drawing on sources where at least three different studies were reported. At the top of the list is clofazimine. Based on five reported values, the standard deviation is 0.93 log unit. Similarly, even though terfenadine solubility has been reported in eleven studies, its interlaboratory reproducibility at 0.71 is still high. Still, 32 out of 3014 molecules is a small fraction. The rest of the molecules are much better determined. Absent from the list in Table 1 is diclofenac, because its intrinsic solubility has a tight consensus value. Its averaged log *S*_0_^25^ = -5.34 ±0.18, based on 34 published studies. The standard deviation is almost identical to the estimated interlaboratory reproducibility of 0.17. Thus, the diclofenac data from different laboratories offer an opportunity to examine issues that affect the quality of solubility measurements as a whole. That is, many of the possible challenges in the interpretation of data in the assessment of interlaboratory reproducibility can be revealed in the examination of diclofenac.

It is evident that several different patterns of distortion occur in the diclofenac log *S-*pH profiles in Figure 9 which cannot be predicted by the Henderson-Hasselbalch equation.

One case, in Figure 9g, shows distortion similar to those shown in Figure 5, suggestive of charge-species aggregation reaction. The data can be rationalized by the formation of the hexameric diclofenac anion complex associated with three protonated 1-(2-hydroxyethyl)pyrrolidine cations. The points with the highest solubility (above the solid curve) appear to be indicative of supersaturation, and could not be explained by a simple equilibrium model.

Distortions involving the free acid are evident in Figures 9f-o, which show elevated solubility in low-pH region, above that indicated by the dashed HH-calculated curve. These may indicate amorphous phase solubility (cf., Figure 1a,d). In some cases, the effect may be due to the limit of detection in the analytical technique used to measure the concentration of the practically-insoluble drug.

Different solubility methods were used to collect the data in the figures. Figure 9a is based on HTS data (μSOL method) – there are many points in the plot, but they show considerable scatter. The rest of the examples are based on shake-flask measurement. Also, the temperature is not the same in the figure. Two of the studies were done at 22 °C, four at 23 °C (‘room temperature’), ten at 25 °C, and one at 37 °C.

The rigorous regression analysis of the data in the figures, taking into account all relevant reactions in the equilibrium model [22], produced the intrinsic solubility values indicated in each frame of Figure 9. When these values are further transformed to a common reference temperature of 25 °C [23], the resultant mean value of the 17 cases turns out to be log *S*_0_^25^ = -5.30 ±0.17 (1.6 ± 0.6 μg/mL). This mean is essentially the same as the one obtained from the averaging all 34 cited values, as noted above. The above standard deviation of the mean is identical to the interlaboratory reproducibility obtained from the analysis of 870 different molecules, and is quite a bit lower than the frequently cited uncertainty of ~0.7 log unit in solubility measurement, when comparing results from different laboratories [25].

How does such an impression of poor reproducibility in solubility measurement arise? As mentioned earlier, it is not uncommon to assume that the solubility 2 pH units below the p*K*_a_ (for acids) or above the *pK_a_* (for bases) can be equated with the intrinsic solubility. Clearly, such an approximation would not be accurate in cases of the distortions found in Figures 9f-o (and those in Fig. 1a,d). The consequences of the above approximation can be illustrated by selecting the lowest solubility in each of the 17 cases and calculating the average: log *S*_0_^25^ (approx.) = -5.01 ±0.59 (6.5 ± 8.5 μg/mL). The increased apparent standard deviation, 0.59, seems to be consistent with the ~0.7 value.

### Prediction of intrinsic solubility

Many computational methods for predicting solubility have been described. These serve the important need to predict solubility risks during structural design in the early stages of drug discovery. The earlier methods used relatively small training sets (<1000 compounds), consisting of molecules (including liquids and gases) such as alcohols, pesticides, and herbicides. This limited the accuracy of the prediction of the solubility of drug solids. As more drug data became available for training the prediction models, methods improved. The ‘solubility challenge’ posed by Llinàs *et al.* [65,66] spurred fresh discussion about prediction efficacy and the quality of available data. The meta-analysis described in the preceding section examined factors related to data quality [50]. The study suggested two ways to improve quality of legacy measurement of equilibrium solubility, as discussed above. Methods based on shake-flask measurement as a function of pH (‘gold standard’), and two potentiometric methods (*p*SOL & CheqSol) can be recommended [4]. Predictions of solubility in simulated and aspirated intestinal fluids are exciting new topics in current research [67].

## Summary and conclusions

This review, albeit brief, points towards the complexity in understanding and predicting solubility-related processes. These include dissolution, solubilization/aggregation, pH-, counterion- and buffer strength effects on solubility, and the impact of salt and cocrystal formation on dissolution, solubility and supersaturation. The debate around how consistent data we can achieve from solubility assays and to what extent the data can be used to model and predict, *e.g*., pH-dependence or solubilization tendencies in different solvents will continue. A platform for such a vibrant discussion which will spur new ideas is the upcoming IAPC-8 meeting where these topics will be further explored.

## Figures and Tables

**Table 1.** Averaged intrinsic solubility of 32 drugs at 25 °C with the *poorest* interlaboratory reproducibility

Compound	log *S*_0_^25^ (M)	SD ^a^	n ^b^	Compound	log *S*_0_^25^ (M)	SD	n
Clofazimine	-9.05	0.93	5	Amiodarone	-10.41	0.59	4
Telmisartan	-6.73	0.84	5	Saquinavir	-5.92	0.58	3
Buprenorphine	-6.07	0.83	3	Quinine	-3.06	0.57	7
Mifepristone	-5.22	0.75	4	Diflunisal	-4.99	0.56	11
Tamoxifen	-7.52	0.72	7	Raloxifene	-6.82	0.56	6
Terfenadine	-7.74	0.71	11	Diphenhydramine	-3.21	0.55	4
Sulfadimethoxine	-3.74	0.70	3	Etoxadrol	-1.96	0.55	3
Curcumin	-5.36	0.68	3	Didanosine	-1.24	0.54	3
Rifabutin	-4.09	0.66	3	Danazol	-6.10	0.52	10
Iopanoic_Acid	-5.49	0.66	3	Ezetimibe	-4.94	0.51	4
Pioglitazone	-6.20	0.66	4	Chlorprothixene	-5.99	0.51	6
Amodiaquine	-5.49	0.65	3	Bromocriptine	-5.50	0.51	5
Fentiazac	-5.84	0.65	4	Miconazole	-5.82	0.50	6
Itraconazole	-8.98	0.61	3	Omeprazole	-3.70	0.50	3
Procaine	-2.30	0.60	3	Amantadine	-2.19	0.50	3
Bisoprolol	-2.09	0.59	3	Thiabendazole	-3.97	0.50	4

^a^ Calculated interlaboratory standard deviation.

^b^ Number of reported studies from different laboratories.
